# Scaling up Rural Sanitation in India

**DOI:** 10.1371/journal.pmed.1001710

**Published:** 2014-08-26

**Authors:** Clarissa Brocklehurst

**Affiliations:** 1 Department of Environmental Sciences and Engineering, Gillings School of Global Public Health, The University of North Carolina at Chapel Hill, Chapel Hill, North Carolina, United States of America; 2 Sanitation and Water for All Secretariat, New York, New York, United States of America

## Abstract

Clarissa Brocklehurst discusses the study by Patil and colleagues on a sanitation program in India and highlights the challenges ahead to improve the situation for millions of people who still have no option but to practice open defecation.

*Please see later in the article for the Editors' Summary*

**Linked Research Article**

This Perspective discusses the following new study published in *PLOS Medicine*:

Patil SR, Arnold BF, Salvatore AL, Briceno B, Ganguly S, et al. (2014) The Effect of India's Total Sanitation Campaign on Defecation Behaviors and Child Health in Rural Madhya Pradesh: A Cluster Randomized Controlled Trial. PLoS Med 11(8): e1001709. doi:10.1371/journal.pmed.1001709

Sumeet Patil and colleagues conduct a cluster randomized controlled trial to measure the effect of India's Total Sanitation Campaign on the availability of individual household latrines, defecation behaviors, and child health in Madhya Pradesh.

The WHO-UNICEF Joint Monitoring Program (JMP) for Water and Sanitation, which tracks progress towards the water and sanitation targets of the Millennium Development Goals, estimates that 36% of the world's population, or 2.5 billion people, lack access to an improved sanitation facility, defined by the JMP as “one that hygienically separates human excreta from human contact” [1]. This situation means that a large proportion of the world's people live at risk of contamination of their environment by human fecal matter.

Even more striking is the number of people who resort to the euphemistically-named practice of “open defecation”, defined by the JMP as “no facilities or bush or field includes defecation in the bush or field or ditch; excreta deposited on the ground and covered with a layer of earth (cat method); excreta wrapped and thrown into garbage; and defecation into surface water (drainage channel, beach, river, stream or sea)” [2]. Over 1 billion people still practice open defecation, and this sanitation practice has been associated not only with conditions such as diarrhea and helminth infections but also with stunting in children [3],[4].

The situation is particularly acute in India, in which 64% of the population does not use improved sanitation. But of even more concern is the fact that almost half of people living in India practice open defecation. In rural India, the proportion of people practicing open defecation is even higher, at 66% [1]. India has the questionable distinction of being the country with largest number of people practicing open defecation in the world: 597 million people, more than ten times the number of any other single country. Globally, more than half of the people who practice open defecation live in India [1].

This dire situation has its consequences. It is estimated that poor sanitation and the practice of open defection have disastrous impacts on the health of India's population, and on the country's economy. The Water and Sanitation Program of the World Bank estimates that a lack of sanitation costs India US$48 per person per year, the equivalent of 6.4% of the country's gross domestic product (GDP) [5]. Open defecation is increasingly linked to India's particular problem with stunting among its children, which perplexingly endures at levels higher than sub-Saharan Africa, despite increasing prosperity in the country [6],[7].

The real human cost of poor sanitation, though, cannot be more vividly illustrated than by the recent incident in which two girls were raped and murdered in the Indian state of Uttar Pradesh while searching for a private place to defecate [8]. While this story made international headlines, the fact is that many women face harassment, assault, and rape when seeking some measure of privacy. This situation makes a mockery of the fact that in 2010 sanitation was recognized by the member states of the United Nations as a human right [9].

In this context, the Government of India launched its ambitious Total Sanitation Campaign. But a paper published in this week's issue of *PLOS Medicine* by Patil and colleagues shows that improving sanitation in rural India is far from straightforward [10]. Patil and his co-authors describe the outcomes of a Randomized Control Trial (RCT) to examine the outcomes of a major government-led rural sanitation campaign in the Indian state of Madya Pradesh. The authors assess the progress the program made in terms of increasing improved sanitation as defined by the JMP, in reducing open defecation, and in improving the health of children. This study is very important given the relative paucity of rigorous research on the outcomes of sanitation programs, especially those at this kind of scale.

It is disappointing that the authors find that the program resulted in very limited increases in adoption of improved sanitation and had even less impact on the practice of open defecation. The findings suggest that a significant number of families that constructed latrines under the program, incentivized by hardware subsidies, actually continued to practice open defecation. Furthermore, the modest increases in the use of improved sanitation did not result in improvements in child health [10].

It might be tempting to conclude from these findings that sanitation is not a good investment and that interventions at this scale are doomed to fail. That would be a mistake—the study shows simply that sanitation in rural areas is hard to get right. There are passionate debates in the water and sanitation sector about the best way to approach sanitation, which is, after all, more a question of behavior than of bricks and mortar. This study certainly illustrates this reality, and it shows that open defecation is a persistent behavior, practiced even by those with latrines at home. It suggests that, no matter how generous, subsidies designed to pay for the construction costs of latrines will not, of themselves, result in significantly increased use of latrines. It also implies that small, incremental improvements in sanitation are not enough—conventional wisdom in the sanitation sector has it that a “tipping point” is needed before latrine use becomes a social norm in a community. Creating this new norm is also important from a health perspective; greater reductions in the practice of open defecation, and thus reductions in the extent of fecal contamination of the places where people live, are needed before significant health improvements are likely to be achieved.

Improving sanitation globally is an imperative, one that is nowhere more urgent than in India. The fact that it is hard to do and prone to failure is no reason to lose heart or to give up. Unfortunately, while RCTs may tell us about the outcomes of a program such as the one studied by Patil and his colleagues [10], the type of information they gather does not tell us enough about the success or failure of the programmatic approaches used, meaning that they can only give insight into the challenges lying ahead; in this case, the challenge that even a program with significant investment may not result in the expected outcomes. A detailed examination of the actions of 51 districts in 12 Indian states implementing the Total Sanitation Campaign has been carried out by the World Bank Water and Sanitation Program [11]. Although the researchers did not have access to the detailed and robust outcome data that an RCT produces, and they did not examine health impacts, the researchers scored actions undertaken by district governments and compared them with available government data on toilet usage. Their report concluded that districts in which the focus was on changing collective behavior, creation of demand for sanitation, and the development of technological solutions tailored to consumer preferences had the most success in increasing use of toilets [11].

There is an urgent need to continue to expand global understanding of what works, as well as what does not work, and keep focused on the important task of winning the sanitation battle.

And a battle it is, certainly in India, where not only are there hundreds of millions of people practicing open defecation but also new analysis of household survey data shows that the density of open defecation, measured as people defecating in the open per square kilometer, is increasing [12]. There is no time to be lost. If generations of children are to be saved from the stunting and ill-health that poor sanitation causes, and generations of women and girls are to be saved from the indignity and risk that open defecation entails, then addressing sanitation must be one of India's highest priorities.

## References

[1] WHO-UNICEF (2014) Progress on Drinking Water and Sanitation: 2014 update. Available: http://www.unicef.org/media/files/JMP_2014_Update.pdf. Accessed 21 July 2014.

[2] WHO-UNICEF (2014) Improved and unimproved water and sanitation facilities. Available: http://www.wssinfo.org/definitions-methods/watsan-categories/. Accessed 30 July 2014.

[3] Humphrey JH (2009) Child undernutrition, tropical enteropathy, toilets, and handwashing. Lancet 374: 1032–1035 doi:10.1016/S0140-6736(09)60950-8 19766883 10.1016/S0140-6736(09)60950-8

[4] Dangour AD, Watson L, Cumming O, Boisson S, Che Y, et al (2013) Interventions to improve water quality and supply, sanitation and hygiene practices, and their effects on the nutritional status of children. Cochrane Database Syst Rev 8: CD009382 doi:10.1002/14651858.CD009382.pub2 10.1002/14651858.CD009382.pub2 PMC11608819 23904195

[5] Water and Sanitation Program (WSP) (2010) The Economic Impacts of Inadequate Sanitation in India. Available: http://www.zaragoza.es/contenidos/medioambiente/onu/765-eng.pdf. Accessed 21 July 2014.

[6] Spears D (2013) The nutritional value of toilets: How much international variation in child height can sanitation explain? RICE Working Paper. Available: http://riceinstitute.org/wordpress/wp-content/uploads/downloads/2013/07/Spears-height-and-sanitation-6-2013.pdf. Accessed 21 July 2014.

[7] Harris G (2014 July 13) Poor Sanitation in India May Afflict Well-Fed Children With Malnutrition. The New York Times Available: http://www.nytimes.com/2014/07/15/world/asia/poor-sanitation-in-india-may-afflict-well-fed-children-with-malnutrition.html?_r=0. Accessed 21 July 2014.

[8] Soutik B (2014 May 30) Why India's sanitation crisis kills women. BBC News India Available: http://www.bbc.com/news/world-asia-india-27635363. Accessed 21 July 2014.

[9] United Nations General Assembly (2010) Resolution 64/292 adopted by the General Assembly on 28 July 2010: The human right to water and sanitation. Available: http://www.un.org/ga/search/view_doc.asp?symbol=A/RES/64/292. Accessed 21 July 2014.

[10] Patil SR, Arnold BF, Salvatore AL, Briceno B, Ganguly S, et al (2014) The Effect of India's Total Sanitation Campaign on Defecation Behaviors and Child Health in Rural Madhya Pradesh: A Cluster Randomized Controlled Trial. PLoS Med 11: e1001709. 25157929 10.1371/journal.pmed.1001709 PMC4144850

[11] Water and Sanitation Program (WSP) (2013) Linking Service Delivery Processes and Outcomes in Rural Sanitation: Findings from 56 Districts in India. Available: http://www.wsp.org/sites/wsp.org/files/publications/WSP-Linking-Service-Delivery-Processes-Outcomes-Rural-Sanitation-Findings-Districts-India.pdf. Accessed 21 July 2014.

[12] Spears D (2014) Increasing average exposure to open defecation in India, 2001–2011. Rice Institute Available: http://riceinstitute.org/wordpress/wp-content/uploads/downloads/2014/06/Spears-2014-increasing-OD-density.pdf. Accessed 21 July 2014.

